# Host genetic factors associated to proviral load in Peruvian HTLV-1 infected

**DOI:** 10.1186/1742-4690-8-S1-A147

**Published:** 2011-06-06

**Authors:** Michael Talledo, Giovanni López1, Jeroen R Huyghe, Kristien Verdonck, Elsa González, Martin Tipismana, Daniel Clark, Guido Vanham, Eduardo Gotuzzo, Guy Van Camp, Lut Van Laer

**Affiliations:** 1 Inst de Med Trop Alexander von Humboldt, Univ. Peruana Cayetano Heredia, Lima, Peru; 2 Dept of Med Genetics, Univ. of Antwerp, Antwerp, Belgium; 3 Virology Unit, Dept. of Microbiol., Inst. of Tropical Med., Antwerp, Belgium; 4 Fac de Ciencias y Filosofía Lab de Inves y Des, Univ. Peruana Cayetano Heredia, Lima, Peru; 5 Dept of Biomedical Sci., Univ. of Antwerp, Antwerp, Belgium

## Introduction

Patients with HTLV-1 associated myelopathy (HAM/TSP) tend to present higher HTLV-1 proviral load (PVL) than asymptomatic carriers (AC). PVL remains the only marker consistently associated with HAM/TSP across several populations. We report a candidate gene study to identify associations between host genetic markers and PVL.

## Subjects and methods

170 HTLV-1 positive individuals were analyzed (56 HAM/TSP and 114 AC). Six HLA alleles and 140 SNPs belonging to 40 candidate genes were genotyped, with 36 ancestry informative markers included into the analysis to correct by population stratification. PVL was determined by a SYBR Green-based realtime quantitative PCR using ERV-3 as reference gene.

The effect of each SNP was evaluated with linear regression analysis. Disease status, age, gender, and the first three principal components were included as covariates.

Due to the exploratory nature of the study, no correction for multiple testing was performed; a P value<0.05 was considered significant.

## Results

After multivariate linear regression analysis, two SNPs: IL6–174 (Interleukine 6) (P=0.0013) and ICAM1-R241G (Intercellular Adhesion Molecule 1) (P=0.0084), and HLA-A*02 (P=0.0306) showed association with PVL.

## Conclusions

These results suggest a possible implication of IL6–174, ICAM1-R241G and HLA-A*02 allele in the PVL among Peruvian HTLV-1 infected individuals. IL-6 promotes T cell activation, growth, and differentiation of cytotoxic T lymphocytes; ICAM-1 was reported as cofactor for HTLV-1-induced cell fusion and is upregulated in HTLV-1 infected cells; a protective effect associated with TAX has been suggested for HLA-A*02. A replication study in an independent population is needed to confirm our findings.

